# Towards to understanding the preliminary loss and absorption of nitrogen and phosphorus under different treatments in cotton drip- irrigation in northwest Xinjiang

**DOI:** 10.1371/journal.pone.0249730

**Published:** 2021-07-21

**Authors:** Honghong Ma, Shenghai Pu, Pan Li, Xinxiang Niu, Xianglin Wu, Zhiying Yang, Jingrong Zhu, Tao Yang, Zhenan Hou, Xingwang Ma

**Affiliations:** 1 Agricultural College of Shihezi University, Shihezi, Xinjiang, China; 2 Institute of Soil, Fertilizer and Agricultural Water Conservation, Xinjiang Academy of Agricultural Sciences, Urumqi, Xinjiang, China; 3 Key Laboratory of Agri-Environment of Northwest Oasis, Ministry of Agriculture and Rural Affairs, Urumqi, Xinjiang, China; 4 Institute of Agricultural Quality Standards and Testing Technology, Xinjiang Academy of Agricultural Sciences, Urumqi, Xinjiang, China; Soil and Water Resources Institute ELGO-DIMITRA, GREECE

## Abstract

Drip irrigation under plastic mulch is widely used in Xinjiang, Northwest China. It can not only save water, but also reduce nutrient loss and improve fertilizer utilization. However, it is not clear whether the leaching occurs or not, what is the leaching amount? What is the relationship among fertilization, irrigation regimes, loss, cotton absorption, and cotton field under different fertilization and irrigation management under drip irrigation? Studying these issues not only provides reference for the formulation of fertilization and irrigation systems, but also is of great significance for reducing non-point source pollution. A long-term positioning experiment was conducted from 2009 to 2012 in Baotou Lake farm in Korla City, Xinjiang, with drip-irrigated cotton (*Gossypium hirsutum* L.) under different N fertilizer and irrigation amounts. The treatments were designed comprising Control (CK,0 N, 0 P, and 0 K with an irrigation of 480 mm) and the following three other treatments: (1) Conventional fertilize and irrigation (CON, 357 kg N hm^–2^, 90 kg P hm^–2^, 0 kg K hm^–2^, and irrigation of 480 mm); (2) Conventional fertilization and Optimizing irrigation (OPT, 357 kg N hm^–2^, 90 kg P hm^–2^, 62 kg K hm^–2^, and irrigation of 420 mm); and (3) Optimizing fertilization and irrigation (OPTN, 240 kg N hm^–2^, 65 kg P hm^–2^, 62 kg K hm^–2^, and irrigation of 420 mm). The results found that the leaching would occur in arid area under drip irrigation. The loss of total N, NH_4_^+^, P, N and P loss coefficient was higher under conventional fertilize and irrigation treatment while the loss of NO_3_^-^ was higher under conventional fertilization and optimizing irrigation treatment. The correlations among N, P absorption by cotton, loss of NH_4_^+^ and total phosphorus were quadratic function. The total nitrogen loss and cumulative nitrogen application was lineally correlated. The loss of NO_3_^-^ and cumulative nitrogen application was exponential. The nitrogen and phosphorus absorption by cotton under conventional fertilization and optimizing irrigation treatment was 24.53% and 35.86% higher than that in conventional fertilize and irrigation treatment, respectively. The cotton yield under conventional fertilization and optimizing irrigation treatment obtained higher than that in other three treatments. Therefore, the conventional fertilization and optimizing irrigation treatment was the optimal management of water and fertilizer in our study. These results demonstrate that reasonable water, nitrogen and phosphorus fertilize could not only effectively promote the absorption of nitrogen and phosphorus, but also reduce nitrogen and phosphorus losses under drip fertigation and plastic mulching.

## Introduction

In recent decades, non-point source pollution (NPSP) has become a major threat to the water quality of global water resources [1–3]. Non-point source pollution (NPSP) is influenced by lots of factors, such as soil type, land use, climate, hydrology, and management [4]. Compared with point source (PS) pollution, non-point pollution is not easily controlled because of its diffuse sources, which usually comes from agricultural activities [5]. Total nitrogen (TN) and total phosphorus (TP) loads are regarded as the main two indexes in water quality assessment in China [6,7]. Nitrogen (N) and phosphorus (P) generally come from fertilizers in the farmland. Influenced by the traditional ideas of farmers, it is considered that the yield of crops with more fertilizer and water is higher. Thus excessive application of nitrogen (N), phosphorus (P) being added to aquatic environments, which causes a series of problems such as eutrophication of water bodies, lower soil productivity, and poor quality water for drinking and other purposes [8–12]. Most studies of soil and nutrient losses have been done. And they were mainly focused on the surface runoff [13,14]. However, the soil and nutrient losses in the underground leaching water by drip irrigation are less.

The autonomous province of Xinjiang in north-western China is an arid region in which the main cash crop is cotton (*Gossypium hirsutum* L.) [15]. Due to the shortage of water resources in Xinjiang, drip irrigation is widely used in cotton. Drip fertigation can minimize evaporation loss, allow the amount of water and the concentrations of nutrients supplied to crops to be precisely controlled, thereby conserving water and reducing fluctuation in the concentrations of nutrients in soil during the growing season, resulting in increasing yield of cotton [16,17].

Nitrogen and Phosphorus are essential plant nutrients. Nitrogen is essential for plants and is also an important limiting factor to soil productivity. Mineralization converts organic N into NH_4_^+^ and nitrification converts NH_4_^+^ into NO_3_^-^, which are absorbed and used by crops and constitute what is termed available N [18]. Nitrate is highly mobile and leachable. It has been established that excessive application of N leads to nitrate pollution of groundwater and surface water [19,20]. Phosphorus can be transported to water bodies via storm run-off in dissolved and particulate forms [2]. Reasonable water and N and P need to be supplied in the right amounts for higher crop yields [21]. However, improper use of fertilizers could lower soil fertility, transport N and P from farmlands into aquatic systems [22], then reduce crop productivity and water body pollution.

Therefore, it is necessary to estimate and control the loss of N and P to minimize the pollution of surface water [23]. Verifying the relationships between application of fertilizers and loss of nutrients is the first step in reducing NPSP [24]. Earlier studies found the relationship between N application and N loss to be inconsistent: it could be linear, exponential, or quadratic [25–29]. Continued and long-term application of fertilizers is believed to lead to accumulation of P in soils, and in turn, resulting in greater loss of P from soil to surrounding aquatic ecosystems [30]. These studies are biased towards diffuse irrigation. However, it relates to drip irrigation has received little attention. In addition, the relationship between nitrogen, phosphorus absorption of cotton and nitrogen, phosphorus leaching forms is still unclear, especially under four traditional fertilizer in Southern Xinjiang under drip irrigation.

The objectives of this study were: (1) to define the characteristics of nitrogen and phosphorus loss under different treatments under drip irrigation in arid area; (2) to verify which relationship between the accumulation of nitrogen and phosphorus in soil and plant absorption under drip irrigation in arid area; (3) which treatment had less nitrogen and phosphorus loss and higher yield under drip irrigation in arid area. The results of the study will be particularly helpful in choosing fertigation regimes to minimize the leaching of N and P and to boost higher yields.

## Materials and methods

### Site description and soil properties

Experiments were conducted in each cotton-growing season from 2009 to 2012 in different fields that are part of the Baotou Lake farm in Korla City, Xinjiang, Northwestern China (41°40′48′′N, 85°40′12′′E). The climate is continental arid, with average annual precipitation of 56.2 mm and potential evaporation of 2497.4 mm. The accumulated temperature is 4252.2°C and the frost-free period is 205 days. The groundwater level is 2 m to 2.5 m. The soil is a sandy loam and moderately fertile. The bulk density of surface soil (0–20 cm) in the field was 1.23 g cm^−^^3^. In 2009, the properties of surface soil at the trial site were as follows: pH, 8.46; organic matter, 7.51 g kg^−1^ (estimated by the wet oxidation method); total Kjeldahl N, 0.45 g kg^−1^; total Kjeldahl P, 0.046 g kg^-1^; 0.5 M NaHCO_3_ extractable P, 4.99 mg kg^-1^; 0.01 M CaCl_2_ extractable P, 1.87 mg kg^-1^; available potassium (K), 95.93 mg kg^-1^; NO_3_^–^-N, 7.27 mg kg^-1^; and NH_4_^+^-N, 3.61 mg kg^-1^.

### Experimental design and agronomic management

All the experiments were laid out in a factorial design. The control consisted of 480 mm of irrigation without any fertilizers (CK, no fertilization and irrigation). The treatments comprised varying doses of N, P, and K and varying amounts of irrigation. N was given in the form of urea; P, as calcium phosphate; and K, as potassium sulphate. The treatments were as follows: (1) Conventional fertilization and irrigation (CON, 357 kg hm^-2^ N; 90 kg hm^-2^ P; no K; and irrigation, 480 mm); (2) Conventional fertilization and Optimizing irrigation (OPT, 357 kg hm^-2^ N; 90 kg hm^-2^ P, 62 kg hm^-2^ K; and irrigation, 420 mm); and (3) Optimizing fertilization and irrigation (OPTN, 240 kg hm^-2^ N; 65 kg hm^-2^ P; 62 kg hm^-2^ K; and irrigation, 420 mm)_._ The fertilizers were broadcast manually and then incorporated into soil using a rotator before sowing. Granular urea was applied where required as a band in the rows before sowing as a basal dose, and the remaining N was solubilized and applied in eight separate fertigation events after sowing. Granular urea was applied with 20% of total applied N as a band in the rows before planting the seeds for the three treatments except for CK, and the remaining 80% N solubilized and applied according to the proportion of different treatments after planting (Table 1). For the CON treatment, the remaining 80% N solubilized and applied over the second, third and fourth irrigation events applied 36.8%, 21.6%and 21.6%, respectively. For the OPT treatment, the remaining 80% N solubilized and applied over the second, third, fourth and fifth irrigation events applied 32%, 20%, 16% and 12%. For the OPTN treatment, the remaining 80% N solubilized and applied over the second, third, fourth and fifth irrigation events applied 32%, 20%, 16% and 12%. Details of the fertilizer doses are shown in Table 1. The treatments were laid out in a randomized complete block design with three replications. Each plot was 4.5 m × 7.4 m.

**Table 1:** Fertilizer doses applied per irrigation (kg·hm^-2^).

Treatment	Fertilizer applied before sowing	1st	2nd	3rd	4th	5th	6th	7th	8th	Total N Fertilization doses	Total P Fertilization doses
Date	4.20th	6.8th	6.17th	6.25th	7.5th	7.13th	7.21st	7.29th	8.10th		
CK	0	0	0	0	0	0	0	0	0	0	0
CON	71.4	0	131.376	77.112	77.112	0	0	0	0	357	90
OPT	71.4	0	114.24	71.4	57.12	42.84	0	0	0	357	90
OPTN	48	0	76.8	48	38.4	28.8	0	0	0	240	62

1^st^ represented the amount of N fertilizer in the first irrigation event. 2^nd^ represented the amount of N fertilizer in the second irrigation event. 3^rd^ represented the amount of N fertilizer in the third irrigation event. 4th represented the amount of N fertilizer in the fourth irrigation event. 5^th^ represented the amount of N fertilizer in the fifth irrigation event. 6^th^ represented the amount of N fertilizer in the sixth irrigation event. 7^th^ represented the amount of N fertilizer in the seventh irrigation event. 8^th^ represented the amount of N fertilizer in the eighth irrigation event.

In each of the 4 years, cotton (cultivar Xinluzao 38) was sown in late April. The first drip irrigation began in early June, and the last irrigation ended in mid-August. Four irrigation events happened in July, namely once a week. Drip irrigation happened twice in June and August, respectively. Drip irrigation pipes and plastic mulch were in place before sowing, which was carried out using a custom-built tractor-drawn seeder. Seeds were sown in double rows with a gap of 30 cm between the two rows that formed a pair and a gap of 60 cm between one pair and the next. Within each row, seeds were sown 10 cm apart. The plastic mulch comprised high-density, airtight, transparent polythene film in strips wide enough to cover two double rows. Weeds and pests were controlled using standard management practices, namely by applying herbicides and pesticides.

### Collection of leachate and analysis of NO_3_^–^ and NH_4_^+^ levels in leachate

**Fig 1:**
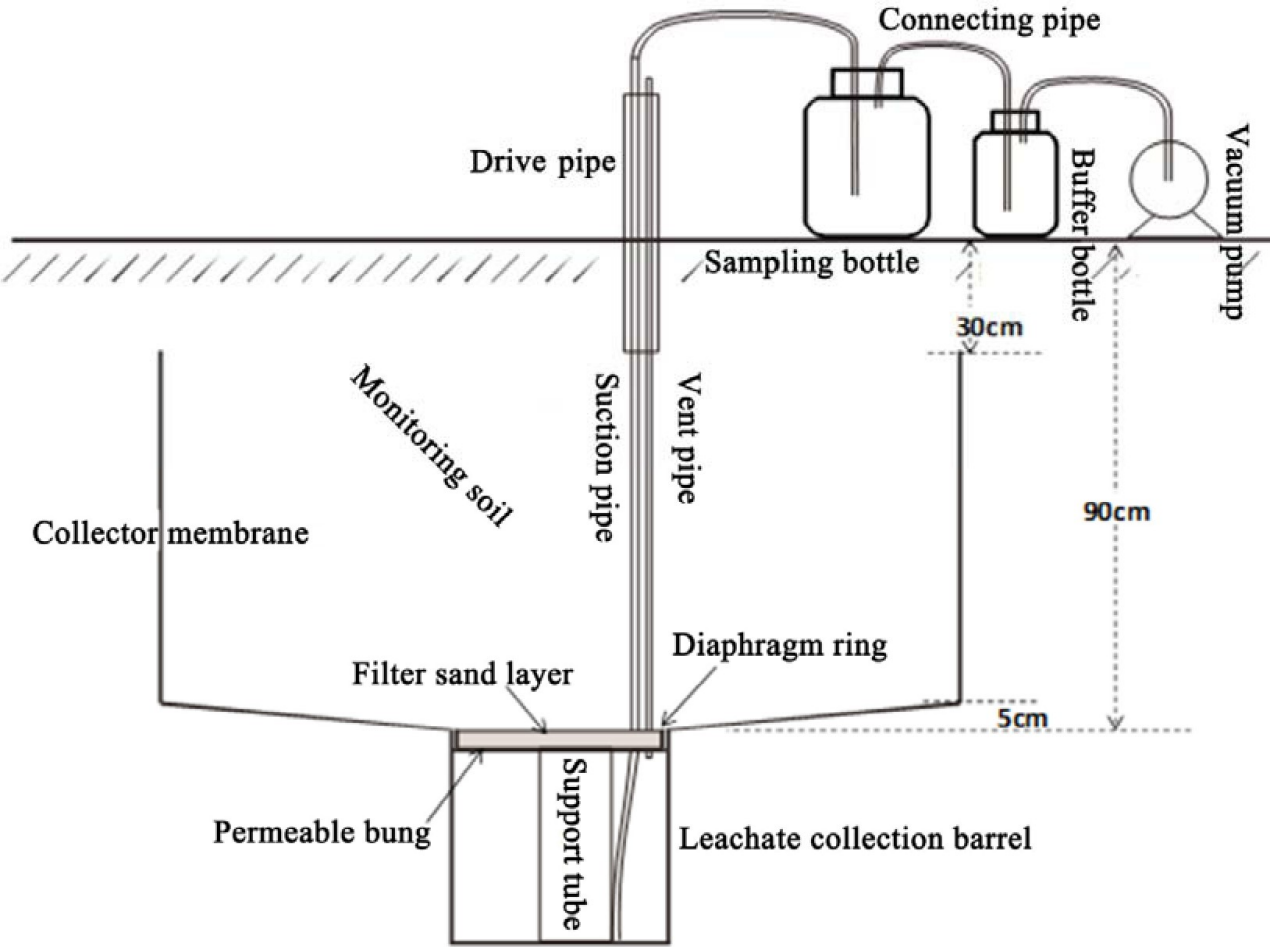
Set-up to collect leachate.

The levels of NO_3_^–^ and NH_4_^+^ were determined in samples of leachate obtained from a drainage collector installed underground. The set-up comprised a special polyvinyl chloride dish (0.30 m long × 0.60 m wide × 0.08 m deep) connected by plastic pipes to a 25 L polyvinyl chloride bucket (Fig 1). A plastic pipe extended from the water storage bucket as a water intake pipe. This assembly was placed at the bottom of a rectangular pit lined on its sides with concrete. The pit, 150 cm long, 80 cm wide, and 90 cm deep, was dug in the middle of the plot. The bottom of the pit was sloped from all sides towards its centre to facilitate the flow of leachate into the collector, which was at the centre of the enclosure. The centre of the pit was dug deeper to accommodate the leaching barrel, which was placed vertically into the round depression. Finally, the rectangular pit was backfilled layer by layer in the reverse order of its excavation and the contents compacted while backfilling. Leachate samples were collected after each round of irrigation.

The leachate volume was measured using a graduated cylinder. Levels of nitrate and ammonium in the leachate were determined by colorimetry, using a continuous-flow analytical system (TrAAcs 2000). Total P in the leachate was determined using the potassium persulfate digestion method [25].

### Plant sampling and analysis

At maturity, different organs of the cotton plant were removed manually from each plot, air dried, and weighed. A 1 m × 2 m area in each plot was harvested manually to determine the total weight of above-ground and underground biomass. The samples for determining N and P concentrations were dried at 70°C and then ground fine enough to pass through a 0.25 mm mesh screen. The samples were digested and analysed for total N using the Kjeldahl method and for total P using vanadium molybdenum yellow colorimetry. Total N and P uptake was calculated as the sum of the product of the weight and N and P concentrations in boll and straw tissues.

### N and P loss analysis

Growing season annual nitrogen loss (L_N_, kg hm^–2^) and N loss coefficient (θ_N_) for each different treatment was calculated as:

Nitrogen loss: $L_{N}=\sum_{i=1}^{m}(c_{i}\times v_{i})$, where L_N_ represents the loss of N, C_i_ represents the concentration of N in the leachate, and V_i_ is the volume of the leachate.

N loss coefficient θ_N_ = (L_N,A_−L_N,CK_)/M_N,A_ × 100, where θ_N_ represents the N loss coefficient, L_N,A_ represents the annual nitrogen loss under A treatment, L_N,CK_ represents the annual nitrogen loss under the control treatment, and M_N,A_ represents the N applied as fertilizer under A treatment.

Growing season annual phosphorus loss and P loss coefficient was calculated exactly the same way as above.

### Statistical analysis

Differences in loss of total N, total P, NO_3_^-^, NH_4_^+^, loss coefficient of N and P, N and P absorption by cotton, cotton yield among four treatments were analyzed by one-way parametric analysis of variance (ANOVA) using SPSS version 18.0. Regression analysis was conducted to examine the relationship between accumulative N and N absorption, accumulative P and P absorption, loss of nitrate N and cumulative N application, loss of ammonium nitrogen and cumulative N application, and accumulative P and total P loss.

The optimal water and fertilizer treatment criteria are as follows:

α = (Y_A_-Y_CK_)/Y_CK_×100, Y_A_ represents the yield under A treatment, Y_CK_ represents the yield under CK treatment. By calculating and comparing α, then obtain the optimal water and fertilizer treatment.

## Results

### Characteristics of nitrogen and phosphorus loss under different treatments

**Fig 2:**
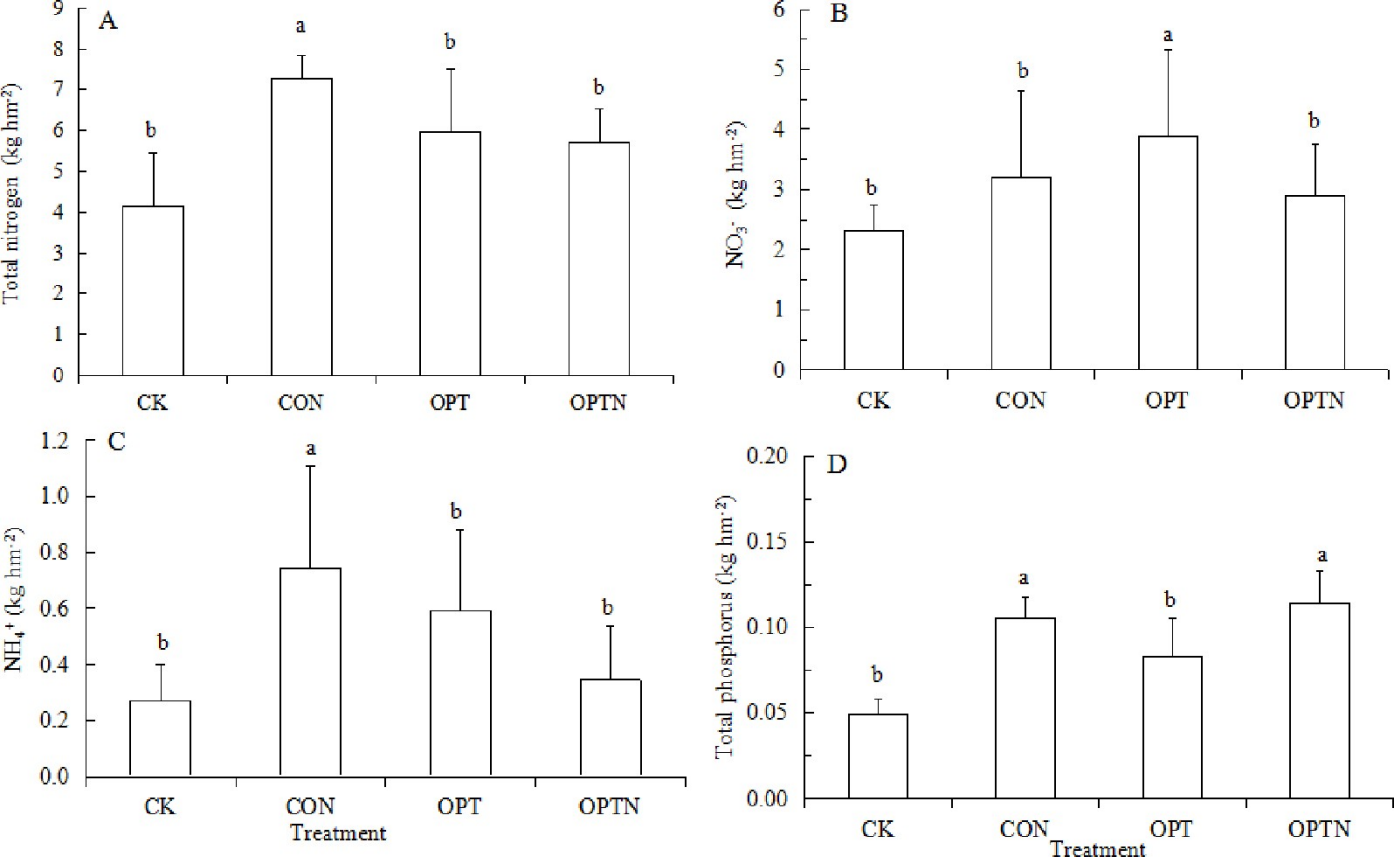
Total N, NO3-, NH4+ and total P loss under four treatments (kg hm^-2^). Data were mean ± standard error (n = 3). Different lowercase letters indicate significant differences among treatments at *P*<0.05 level. Bars mean standard e1rrors.

Total N, NO_3_^-^, NH_4_^+^ and total P loss differed significantly among four treatments. In generally, the leaching loss was more with more fertilization and irrigation. This result can be verified by Fig 2. However, NO_3_^-^ loss in OPT (Conventional fertilization and Optimizing irrigation) treatment was higher than that in CON (Conventional fertilization and irrigation)treatment, probably because the amount of irrigation in OPT treatment was less than that in CON treatment, so the soil temperature was higher in OPT treatment than CON treatment [31], and the transformation of nitrogen fertilizer into soil was faster, NH_4_^+^ was rapidly converted to NO_3_^-^, so the NO_3_^-^ loss in OPT treatment was higher than CON treatment. The loss of nitrogen forms and total P were higher and existed significantly difference under CON treatment compared with other treatments, except for NO_3_^-^ (Fig 2).

The nitrogen loss coefficient were higher under CON treatment, then followed the order: OPT > OPTN (Table 2). The nitrogen loss coefficient could be reduced by 0.50 by reducing the amount of N applied and not changing the amount of irrigation and by 0.29 by reducing the amount of irrigation and not changing the amount of N (Table 2). Clearly, the loss of N through leaching can be reduced effectively by decreasing the amounts of both N and irrigation. For CON and OPT treatment, there was no difference in 2009, the possible reason lies in that the soil fluctuated greatly when the leaching bucket was buried early. From 2010 to 2012, there was significant difference between CON and OPT treatment, which illustrated it was effective measures by decreasing irrigation with the same fertilization.

**Table 2:** Nitrogen and phosphorus loss coefficients, by treatments and growing season (%).

Treatment	Nitrogen loss coefficient				Phosphorus loss coefficient			
	2009	2010	2011	2012	2009	2010	2011	2012
CK	-	-	-	-	-	-	-	-
CON	0.60±0.30a	0.68±0.07a	1.19±0.11a	1.33±0.08a	0.01±0.002b	0.02±0.006a	0.03±0.004a	0.04±0.003a
OPT	0.49±0.06a	0.39±0.05b	0.72±0.06b	0.83±0.02b	0.85±0.095a	0.03±0.005a	0.03±0.003a	0.04±0.003a
OPTN	0.06±0.004b	0.24±0.07b	0.58±0.06b	0.74±0.07b	0.19±0.053b	0.01±0.003a	0.02±0.005a	0.05±0.004a

Data were mean ± standard error (n = 3). Different letters within a column indicate significant differences among treatments by ANOVA followed by Duncan’s test (*p* ≤ 0.05).

The phosphorus loss coefficient showed the same pattern with N. The coefficient could be reduced by 0.05 by reducing the amount of P applied and not changing the amount of irrigation and by 0.01 by reducing the amount of irrigation and not changing the amount of P. Clearly, the loss of P through leaching can be reduced effectively by decreasing the amounts of both P and irrigation (Table 2). There was no difference among treatments from 2010 to 2012 except in 2009, which may be that soil disturbance leaded to the greater P loss.

### Characteristics of nitrogen and phosphorus uptake by cotton under different treatments

The absorption of nitrogen and phosphorus was higher under OPTN treatment in generally. Interestingly, the amount of nitrogen and phosphorus under OPTN treatment was only 240 and 65 kg hm^-2^, respectively, which was obviously lower than that under CON and OPT treatment (360 and 90kg hm^-2^). The absorption of P under OPTN treatment was higher, may be that N fertilizer would affect the absorption of P. And with the increase of nitrogen absorption, the absorption of phosphorus fertilizer increased [32]. These results suggested that the more nitrogen fertilizer plants absorbed more, the less phosphorus fertilizer plants absorbed more under the same irrigation rate.

**Table 3:** Summary statistics of regression analysis of the nitrogen or phosphorus relationship among different treatments.

Treatment	n	Nitrogen			Phosphorus		
		Mean	95% CI	*p*	Mean	95% CI	*p*
CK	12	230.01b	162.47–297.55	<0.01	50.37d	37.53–63.21.08	<0.01
CON	12	530.46a	374.49–686.44	<0.01	88.80c	78.72–98.87	<0.01
OPT	12	543.96a	408.85–679.07	<0.01	122.13b	105.76–138.5082	<0.01
OPTN	12	612.96a	541.30–684.61	<0.01	158.36a	114.12–202.61	<0.01

**Fig 3:**
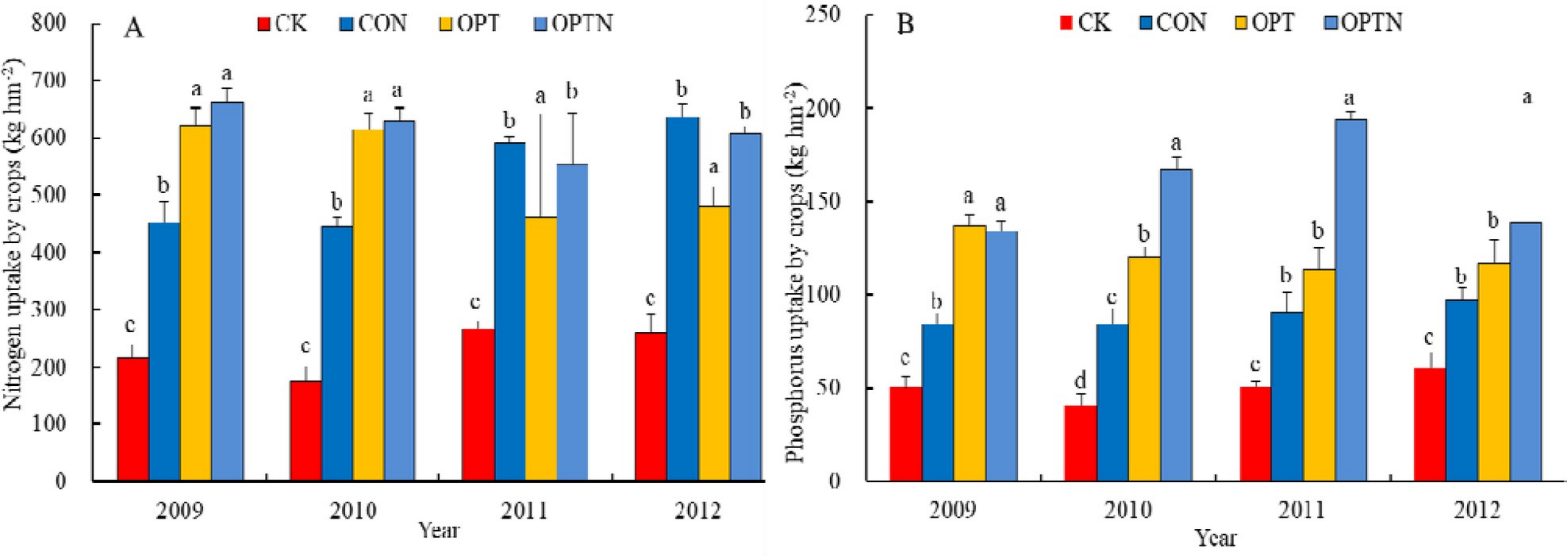
Nitrogen and phosphorus uptake of cotton under four treatments (kg hm^-2^). Note: Data were mean ± standard error (n = 3). Different letters within a column indicate significant differences among treatments by ANOVA followed by Duncan’s test (*p* ≤ 0.05). The corresponding results are presented in Table 3.

Regression results are corresponding to those in Fig 3. n, number of samples; CI, confidence interval. Growth difference of cotton under different treatments is significant when *P*<0.05, vice verse.

### The correlations between nitrogen leaching form, phosphorus leaching form and absorption by cotton and cumulative nitrogen, phosphorus application

**Fig 4:**
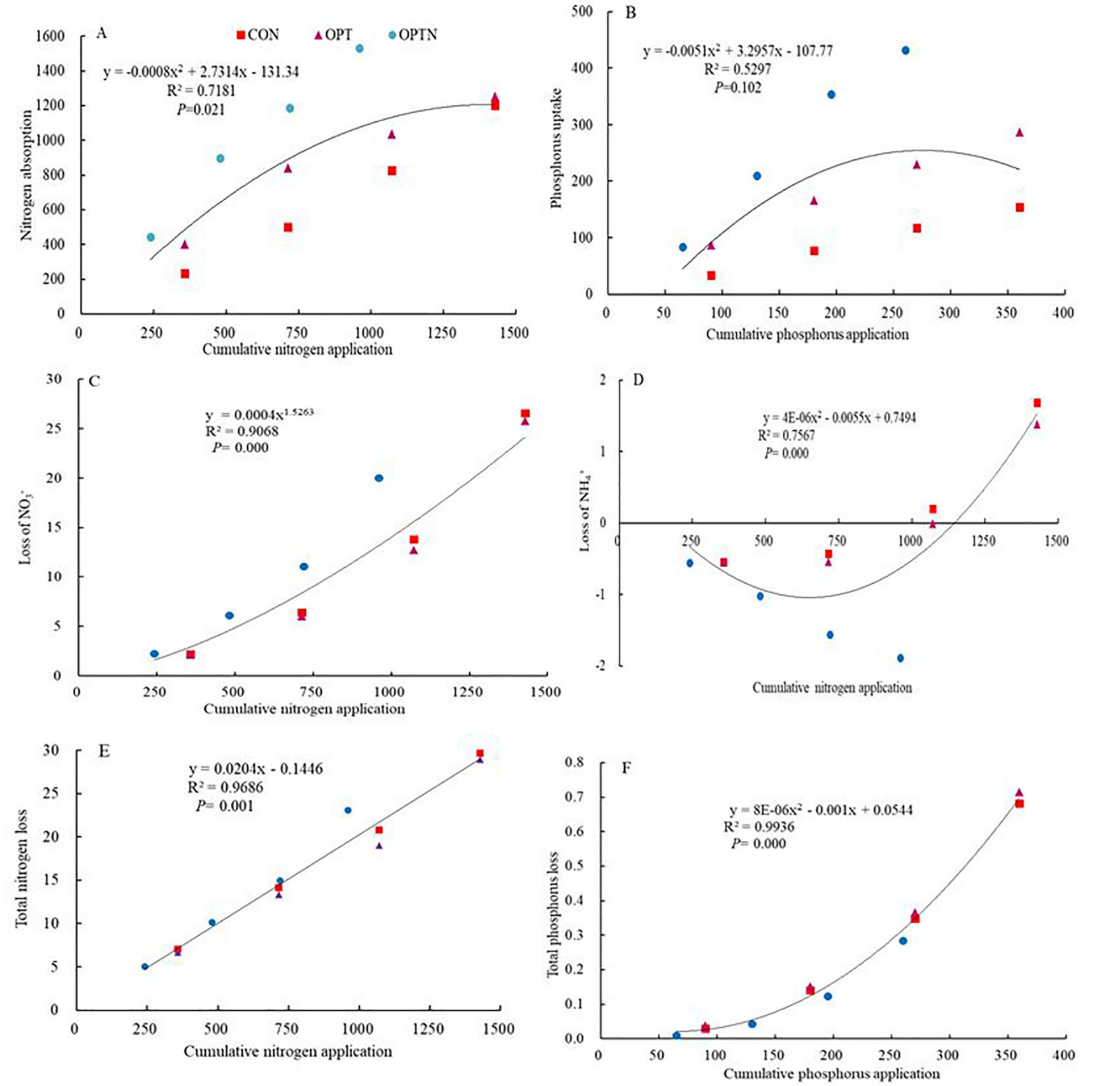
The relationship between nitrogen and phosphorus loss index and nitrogen and phosphorus fertilizer application. The fitting relationship is selected to fit with larger correlation coefficient in the above figure.

The absorption of N and P was quadratic function correlated with the amount of N and P applied (Fig 4A and 4B). Similar relationship applied to the loss of NH_4_^+^ and total phosphorus (Fig 4D and 4F). The relationship between total nitrogen loss and cumulative nitrogen application was lineally correlated (Fig 4E). Moreover, the relationship between loss of NO_3_^-^ and cumulative nitrogen application was exponential (Fig 4C). It is worth noting that the relationships among cumulative nitrogen and phosphorus fertilization, the loss of N and P, the absorption of N and P showed increased trend, but the loss of NH_4_^+^ decreased at beginning, then increased. There was a significant correlation between NH_4_^+^ and NO_3_^-^. Total phosphorus uptake by cotton was positively significant correlated with total nitrogen absorption, while negatively significant correlated with NH_4_^+^. Total N was positively significant correlated with NO_3_^-^ (Table 4).

**Table 4:** The correlations between the total nitrogen, total phosphorus absorption by cotton and loss of nitrogen and phosphorus in leaching water (kg hm^-2^).

	CTN	Total N	NO_3_^-^	NH_4_^+^	CTP	Total P
CTN	1					
Total N	-0.342	1				
NO_3_^-^	-0.0308	0.738**	1			
NH_4_^+^	-0.576*	0.756 **	0.643**	1		
CTP	0.596**	-0.409	-0.439	-0.67**	1	
Total P	-0.138	0.431	0.079	0.345	-0.257	1

CTN represents total nitrogen absorption by cotton; CTP represents total phosphorus uptake by cotton

*, ** mean correlation coefficients are significant at the level of 0.05 and 0.01, respectively.

### Optimal treatment

The cotton yield under four treatments followed the order of: OPT(6.71) > CON(6.39) > OPTN(6.26) > CK(2.54) (Table 5).There was significant difference of cotton yield under CK treatment compared with other three treatments, while the cotton yield among three treatment showed no remarkable difference. For the same treatment, there was difference among years possible due to the different cotton growth. The cotton yield under OPT treatment was 164.17 higher than that under CK treatment. Obviously, the optimal treatment in our study was OPT treatment.

**Table 5:** Cotton yield in different years under different treatments (t hm^-2^).

Yield	2009	2010	2011	2012	Average	α
CK	4.98±016Ac	1.78±0.18Cc	1.67±0.14Bb	1.73±0.15Ab	2.54±0.43b	-
CON	5.89±0.21Bb	6.89±0.13Aa	6.36±0.12Aa	6.41±0.13Ba	6.39±0.11a	151.60
OPT	6.44±0.42Ba	7.14±0.23Ba	6.58±0.16ABa	6.66±0.27ABa	6.71±0.11a	164.17
OPTN	6.50±0.19Ba	5.77±0.17Bb	6.18±0.43ABa	6.60±0.15Aa	6.26±0.12a	146.79

Data were mean ± standard error (n = 3). Different capital letters within a column indicate significant differences among years and different lower case within a row indicate among treatments by ANOVA followed by Duncan’s test (*p* ≤ 0.05). α represents the increase percentage of yield compared to that under CK treatment.

## Discussion

### Nitrogen and phosphorus loss

Due to the traditional belief, the higher the amount of N supplied to a crop, the higher the yield, so farmers often use excessive amounts of N in pursuit of greater profits, particularly in China [31,32]. Such excessive application of N leads to N being leached: the greater the amount of N applied as fertilizer, the greater its loss through leaching, mainly in the form of NO_3_^-^ rather than as NH_4_^+^, which can be easily fixed in soil [23]. This observation is confirmed by our results (Fig 2B and 2C). The loss of NO_3_^-^ was higher under OPT treatment while the loss of NH_4_^+^ and total N was higher under CON treatment. This result probably because the different irrigation leaded to the different microenvironment with the same amount of fertilization, the soil temperature was higher under less irrigation, so the conversion of NH_4_^+^ to NO_3_^-^ was faster, the loss of NO_3_^-^ under OPT treatment was higher. The loss of total P under CON and OPTN treatment was higher than other treatment, probably because the amount of P fertilization in CON was 90 kg hm^-2^ and irrigation was 480 mm, so the loss of total P was higher under CON treatment., The concentration of P in leaching was higher, the leaching volume was lower, so the loss of total P under OPTN treatment was close to that under CON treatment. Thus, there was no difference between CON and OPTN treatment. In this study, the concentration of total P was determined, but the other forms of P were not mentioned. Next, we should determine the concentration of other form P to find out the main form of P loss.

For the nitrogen loss coefficient and phosphorus loss coefficient in the study, the values varied from year to year, but there was no significant difference except for the values in 2009. This may because the installation of the leaching device in each of the plot study sites [33,34]. Turtola et al. [33] studied the subsurface runoff for continuous 10 years and found that the share of subsurface runoff contributed 90% in the first year, 70% in the third year. Marianne [35] also found the similar results. This indicates that the leaching loss may be higher or lower some years after installation of a leaching device.

The input of N in the present study was through irrigation water, applied as the basal dose and top-dressed, atmospheric dry and wet deposition, whereas the output of N was in the form of N leached, plant N uptake, N removed from soil, and N lost through the volatilization of urea. The transformation of N in soil is a complex process [36–41]. However, the loss of N supplied through drip irrigation (fertigation) is not obvious and the loss of nitrogen was very small. Thus, drip irrigation and fertilization can effectively reduce the loss of nitrogen. In addition, we focused only on the loss of N through leaching and ignored the residual N and urea volatilization in the present study. However, because these two forms account for a large proportion of N balance, we intend to examine them in future experiments and work out a complete system of N balance in soil and a crop. Clearly, there was a large proportion of nitrogen in the leaching water except for the loss of total nitrogen, nitrate nitrogen and ammonium nitrogen under different treatment, accounting for total N 38%, 46%, 25% and 43%, respectively. Whether this part of nitrogen has effect on plant absorption and utilization or not and what are the specific nitrogen forms need further study. In addition, Steven et al (2002) [39] found that dissolved organic nitrogen accounts for a major part of nitrogen losses from forests, which could help to explain nutrient limitation in forest ecosystems [32]. It is desirable to detect whether this result is suitable for the N balance in the agro-ecosystem. The rate of loss of N was not even throughout the study, especially in the treatment with higher N fertilize and higher amount of irrigation: the loss coefficient of N ranged from 0.60% to 1.33% from 2009 to 2012, depending on the irrigation and fertilizer regime (CON treatment) (Table 2). Taken together, the loss coefficient under CON treatment was higher than other treatment, nearly 44.36% to 90% of OPTN treatment. Thus, OPTN treatment was the optimal water and nitrogen fertilization management in our study.

The components of soil P are P applied through organic and/or inorganic fertilizers, P removed by the crop, and P lost through other routes [42]. In the present study, P was supplied in the form of an inorganic fertilizer, which made leaching likely to be a major route of loss other than that absorbed by the crop. It was therefore necessary to analyse P lost through leaching from the agro-ecosystem, which is transported through soil water and eventually reaches sources of surface water. As Fig 4F shows, the amount of P lost through leaching was strongly correlated to the amount of P supplied through fertilizers, a result contrary to that reported by Hu [36]; probably, P is fixed in soil in substantial amounts, which lowers the amount likely to be lost through leaching, and P is considered to be relatively immobile in soil compared to N. Phosphorus is firmly bound to soil because of the adsorption of P by Fe and Al oxides in acidic soils [37]. Since 1980, fertilizer P added to soils was observed to have been accumulated in soils in China [38–40]. This accumulated P in the soil is a large potential source of P, which may be available to crops, and leaching accounts for only a small part. This result is consistent with that of Wang [38]. Thus, we should connect the accumulated P in soil, the leaching P, the absorption by plant and P fertilizer together to access P balance. This measure can be helpful to support future crop production.

### Effect on nutrient uptake

Both ammonium (NH_4_^+^) and nitrate (NO_3_^−^) are the major inorganic sources of N for plants [43–48]. Both of them have a significant effect on crop growth and must be managed appropriately to maximize plant growth and NUE. Iqbal, A et al (2020) found that the nitrate uptake by plants was higher than ammonium uptake in cotton. Nutrient uptake by cotton tended to increase with the age of the crop no matter the amount of N and P fertilizers was applied [47]. In our study, total N and P uptake differed significantly depending on the ratio and the level of N and P supplied. N and P absorption by cotton was higher under OPTN treatment, probably because the N, P fertilization and irrigation under OPTN treatment are more suitable for cotton growth. In addition, the microbial activity is more active and can provide more N source for cotton absorption [38,39]. The loss coefficient of N and P was higher under the same treatment (Table 2), probably although the combination of a high doses of fertilization and high levels of organic matter promoted crop growth and accelerated absorption of active nutrients, the availability was in excess of what could be absorbed, so the result was being a greater risk of loss [40]. We found a close correlation between application and absorption for both P and N. Absorption was facilitated by the fertilization regime and the amount of irrigation that constituted the treatment OPT (Fig 4A and 4B). This result showed that although N and P exceeded their respective rates of absorption by the crop, proper irrigation could relieve the effect of excessive fertilization to some extent and improve the efficiency of utilization of N and P. Thus optimal supply of water and fertilization was the key to greater absorption and yield. In our study, although the relationships between N and P fertilization and absorption by cotton were positively correlated, the bearing capacity of soil and absorption of plant was certain and there must be a threshold in the soil. Once the threshold was exceeded, more environmental problems would be caused such as soil salinization, soil hardening, so it is an urgent problem to solve and desirable to study.

## Conclusion

Cotton, one of the main cash crops in Xinjiang, Northwest China, is widely adopted drip irrigation under plastic film in arid area. Although drip irrigation could reduce the waste of water resources, it is not clear whether the leaching occurs and the characteristics of nitrogen and phosphorus loss, especially in arid area. Our results showed that the leaching occurred under four treatments in cotton field under drip irrigation. The greatest loss of total N, NH_4_^+^ and total P was under conventional fertilization and irrigation treatment, which was 28.00%, 11.00%, 115.00%, respectively higher than that under optimizing fertilization and irrigation treatment. The loss of NO_3_^-^ was greatest under conventional fertilization and optimizing irrigation treatment, which was higher 34% than that under optimizing fertilization and irrigation treatment. The greatest loss coefficient of N and P was under conventional fertilization and irrigation treatment. Cotton absorbed higher N was under conventional fertilization and optimizing irrigation treatment, and P was under optimizing fertilization and irrigation treatment. The correlations among N, P absorption, loss of NH_4_^+^ and total phosphorus were quadratic function. The total nitrogen loss and cumulative nitrogen application was lineally correlated. The loss of NO_3_^-^ and cumulative nitrogen application was exponential. The conventional fertilization and optimizing irrigation treatment was the optimal water and fertilization management because the cotton yield was highest With the increase of fertilization application, the amount of N and P loss increased during the period of our study, but how does the loss change after long term of fertilization? If the amount of N and P loss continues to increase, will it be harmful to surface or underground body? This study is based on long term fertilization and irrigation experience under drip irrigation to evaluate N and P loss. This study is desirable to study.

## References

[1] Chen HY, Teng Y G, Wang JS. Load estimation and source apportionment of nonpoint source nitrogen and phosphorus based on integrated application of SLURP model, ECM, and RUSLE: a case study in the Jinjiang River, China. Environment Monit Assess, 2013; 185: 2009–2021. 10.1007/s10661-012-2684-z. 22644124

[2] Li ZW, Zhang GH, Yu XX, Liu QJ, Zhang XC. Phosphorus loss and its estimation in a small watershed of the Yimeng mountainous area, China. Environment Earth Science, 2015; 73: 1205–1216. 10.1007/s12665-014-3475-3.

[3] Min JS, Kong XZ. Research progress of agricultural non-point source pollution. J. Huazhong Agric Univ (Social Sci Ed). 2016; 2: 59–66. (in Chinese) 10.13300/j.cnki.hnwkxb.2016.02.009.

[4] Arabi Mazdak, Rao SG, Mohamed MH, Bernard AE. Role of watershed studivision on modeling the effectiveness of best management practices with SWAT. Journal of the American water resources association. 2006; 42: 518–523.

[5] Ongley ED, Zhang XL, Yu T. Current status of agricultural and rural nonpoint source pollution assessment in China. Environment. Pollution. 2010; 158: 1159–1168. doi: 10.1016/j.envpol.2009.10.047 19931958

[6] Jeon JH, Choi DH, Lim KJ. Automatic calibration of stream flow and nutrients loads using HSPF-PEST at the Bochung A watershed. J. Korean Soc. Agriculture. Engineer. 2010; 52: 77–86.

[7] Zhang BL, Cui BH, Zhang SM, Wu QY, Yao L. Source apportionment of nitrogen and phosphorus from non-point source pollution in Nansi Lake Basin, China Environmental Science and Pollution Research. doi: 10.1007/s11356-018-1956-8 29725920

[8] Li XY, Li BM, Tong Q. The Effect of Drying Temperature on Nitrogen Loss and Pathogen Removal in Laying Hen Manure, Sustainability, 2020, 12(1): 1–11. https://doi.org/103390/su12010403.

[9] Pimentel D, Harvey C, Resosudarmo P, Sinclair K, Kurz D, McNair M, et al. Environmental and economic costs of soil erosion and conservation benefits. Science. 1995; 267: 1117–1123. doi: 10.1126/science.267.5201.1117 17789193

[10] Potter KM, Cubbage FW, Blank GB. A watershed-scale model for predicting nonpoint pollution risk in North Carolina Environmental Management. 2004; 34: 62–74. doi: 10.1007/s00267-004-0117-7 15156350

[11] Farenga SJ, Daniel N. Making a community information guide about nonpoint source pollution. Science Scope. 2007; 30: 12–15.

[12] Chen ZD, Zou Q, Cheng BS, Liang ZQ, Effects of nitrogen on distribution of phosphorus and potassium in wheat plants with different fertilizer tolerance Soil and fertilizer in China. 1993; (4): 24–27. (in Chinese).

[13] Fu B, Liu HB, Lu Y, et al. Study on the characteristics of nitrogen and phosphorus emission in the typical agricultural small watershed of plateau lake-a case study of Feng Yu River Watershed J Environment Science 2015; 35(9): 2982–2899. (in Chinese) https://doi.org/1013671/jhjkxxb20141002.

[14] Zhang BL, Cui BH, Zhang SM, Wu QY, Yao L. Source apportionment of nitrogen and phosphorus from non-point source pollution in Nansi Lake Basin China Environmental Science and Pollution Research. 2018; 25: 19101–19113. doi: 10.1007/s11356-018-1956-8 29725920

[15] Ma ZW, Gao XP, Mario T, Kuang WN, Gui DW, Zeng FJ. Urea fertigation sources affect nitrous oxide emission from a drip-fertigated cotton field in northwestern China. Agriculture Ecosystems & Environment. 2018; 265: 22–30. https://doi.org/101016/jagee201805021.

[16] Baryosef B. Advances in fertigation. Advances in Agronomy. 1999; 65: 1–75. https://doi.org/101016/S0065-2113(08)60910-4.

[17] Kuang WN, Gao XP, Gui DW, Tenuta M, Flaten Donald F, Yin MY, et al. Effects of fertilizer and irrigation management on nitrous oxide emission from cotton fields in an extremely arid region of northwestern China. Field Crops Research. 2018; 229: 17–26. https://doi.org/101016/jfcr201809010.

[18] Khalil A, Singh DK, Singh AK, Khanna M. Modelling of nitrogen leaching from experimental onion field under drip fertigation. Agricultural Water Management. 2007; 89: 15–28. https://doiorg/101016/jagwat200612014.

[19] Yang J, Liang JP, Yang GH, Feng YZ, Ren GX, Ren CJ, et al. Characteristics of Non-Point Source Pollution under Different Land Use Types. Sustainability. 2020; 12(5): 2012. 10.3390/su12052012.

[20] Feng J, Hussain HA, Hussain S, Shi C, Cholidah L, Men SN, et al. Optimum Water and Fertilizer Management for Better Growth and Resource Use Efficiency of Rapeseed in Rainy and Drought Seasons. Sustainability. 2020; 12(2): 1–18. https://doi.org/103390/su12020703.

[21] Xu N, Tan GC, Wang HY, Gai XP. Effect of biochar additions to soil on nitrogen leaching microbial biomass and bacterial community structure. European Journal of Soil Biology. 2016; 74: 1–8. https://doi.org/101016/jejsobi201602004.

[22] Wang WL, Liang T, Wang LQ, Liu YF, Wang YZ, Zhang CS. The effects of fertilizer applications on runoff loss of phosphorus. Environment Earth Science. 2013; 68: 1313–1319. https://doi.org/101007/s12665-012-1829-2.

[23] Ding WC, He P, Zhang J, Liu YX, Xu XP, Ullah S, et al. Optimizing rates and sources of nutrient input to mitigate nitrogen phosphorus and carbon losses from rice paddies. Journal of Cleaner Production. 2020; 256:120603. https://doi.org/101016/jjclepro2020120603.

[24] Cui ZL, Yue SC, Wang GL, Meng QF, Wu L, Yang ZP, et al. Closing the yield gap could reduce projected greenhouse gas emissions: a case study of maize production in China. Global Change Biology. 2013; 19(8): 2467–2477. doi: 10.1111/gcb.12213 23553871

[25] Zhou F, Ciais P, Hayashi K, Galloway J, Kim DG, Yang CL, et al. Affiliations expand Re-estimating NH_3_ emissions from Chinese cropland by a new nonlinear model. Environment Science & Technology. 2016; 50: 564–572. https://doi.org/101021/acsest5b03156. 10.1021/acs.est.5b03156 26710302

[26] Hou XK, Zhou F, Leip A, Fu BJ, Yang H, Chen Yan, et al. Spatial patterns of nitrogen runoff from Chinese paddy fields. Agriculture, Ecosystems and Environment. 2016; 231: 246–254. https://doi.org/101016/jagee201607001.

[27] Eagle AJ, Olander LP, Locklier KL, Heffernan JB, Bernhardt ES. Fertilizer management and environmental factors drive N_2_O and NO_3_^-^ losses in corn: A meta-analysis. Soil Science Society of America Journal. 2017; 81: 1191–1202. https://doiorg/102136/sssaj2016090281.

[28] Wang HY, Zhang D, Zhang YT, Zhai LM, Yin B, Zhou F, et al. Ammonia emissions from paddy fields are underestimated in China. Environmental Pollution. 2018; 235: 482–488. doi: 10.1016/j.envpol.2017.12.103 29324377

[29] Yan X, Wei ZQ, Hong QQ, Lu ZH, Wu JF. Phosphorus fractions and sorption characteristics in a subtropical paddy soil as influenced by fertilizer sources. Geoderma. 2017; 295: 80–85. https://doiorg/101016/jgeoderma201702012Get rights and content.

[30] O’Dell JW. Determination of total Kjeldahl nitrogen by semi-automated colorimetry. Methods for the Determination of Metals in Environmental Samples. 1996; 449–463. https://doi.org/101016/B978-0-8155-1398-850025-2.

[31] Ju XT, Xing GX, Chen XP, Zhang SL, Zhang LJ, Liu XJ, et al. Reducing environmental risk by improving N management in intensive Chinese agricultural systems. Proceedings of the National Academy of Sciences of the United States of America. 2009; 106(9): 3041–3046. doi: 10.1073/pnas.0813417106 19223587 PMC2644255

[32] Ma JC, Liu YX, He WT, He P, Haygarth PM, Surridge BWJ, et al. The long-term soil phosphorus balance across Chinese arable land. Soil Use and Management. 2018; 34: 306–315. https://doi.org/101111/sum12438.

[33] Turtola E, Alakukku L, Uusitalo R, Kaseva A. Surface runoff subsurface drainflow and soil erosion as affected by tillage in a clayey Finnish soil. Agric Food Sci Finl. 2007; 16: 332–351.

[34] Uhlen G. Nutrient leaching and surface runoff in field lysimeters on a cultivated soil Nutrient balances Nor. J Agric Sci. 1989; 3: 33–46.

[35] Marianne EB, Frederik B. Soil Tillage and Crop Growth Effects on Surface and Subsurface Runoff, Loss of Soil, Phosphorus and Nitrogen in a Cold Climate. Land. 2021; 10: 77.

[36] Hu BT, Zhang LJ, Yang SH, Chen YD. Research advances on process and monitoring methods of nitrogen and phosphorus loss in paddy fieids. Journal of Ecology and Rural Environment. 2018; 34(9): 788–796. https://doi.org/1011934/jissn1673-4831201809004.

[37] Philippe H. Bioavailability of soil inorganic P in the rhizosphere as affected by root-induced chemical changes: a review. Plant and Soil. 2001; 237: 173–195. https://doi.org/101023/A:1013351617532.

[38] Wang XL, Feng AP, Wang Q, Wu CQ, Liu Z, Ma ZS, et al. Spatial variability of the nutrient balance and related NPSP risk analysis for agro-ecosystems in China in 2010. Agriculture Ecosystems and Environment. 2014, 193: 42–52. https://doi.org/101016/jagee201404027.

[39] Steven S, Perakis Lars O Hedin, Nitrogen loss from unpolluted South American forests mainly via dissolved organic compounds. Nature. 2002; 415(6870): 416–419. doi: 10.1038/415416a 11807551

[40] Kuo YM, Liu WW, Zhao E, Li R, Muñoz-Carpena R. Water quality variability in the middle and down streams of Han River under the influence of the Middle Route of South-North Water diversion project China. Journal of Hydrology. 2019; 569: 218–229.

[41] Li Z, Zhang R, Wang X, Chen F, Lai D, Tian C. Effects of plastic film mulching with drip irrigation on N_2_O and CH_4_ emissions from cotton fields in arid land. Journal of Agricultural Science. 2014; 152: 534–542.

[42] Hou XY, Wang FX, Han JJ, Kang SZ, Feng SY. Duration of plastic mulch for potato growth under drip irrigation in an arid region of Northwest China. Agricultural and Forest Meteorology. 2010; 150: 115–121.

[43] Günther J, Thevs N, Gusovius HJ, Sigmund I, Brückner T, Beckmann V, et al. Carbon and phosphorus footprint of the cotton production in Xinjiang China in comparison to an alternative fibre (Apocynum) from Central Asia. Journal of Cleaner Production. 2017; 148: 490–497.

[44] Øygarden L, Børresen T. Best management practice in Norway to keep good water quality of syrface waters in rural areas In Prediction and Reduction of Diffuse Pollution Solid Emission and Extreme Flows from Rural Areas—Case study of Small Agricultural Catchment, Banasik K Øygarden L Hejduk L Eds, Wydawnictwo SGGW: Warszawe Poland. 2011, 181–202.

[45] Katri R, Tapio S, Kirsti G, Hannu R. Simulated nitrogen leaching nitrogen mass field balances and their correlation on four farms in south-western Finland during the period 2000–2005. Agricultural and Food Science. 2007; 16: 387–406.

[46] Iqbal A, Qiang D, Wang XR, Gui HP, Zhang HH, Pang NC, et al. Nitrogen preference and genetic variation of cotton genotypes for nitrogen use efficiency Journal of the Science of Food and Agriculture. 2020; 100: 2761–2773. doi: 10.1002/jsfa.10308 32020619

[47] Mahmood T, Kaiser WM. Growth and solute composition of the salt-tolerant kallar grass [*Leptochloa fusca* (L) Kunth] as affected by nitrogen source. Plant Soil. 2003; 252: 359–366.

[48] Wang R, Chen L, Chen J, Chen Y, Zhang Z, Wang X, et al. Different nitrate and ammonium ratios affect growth and physiological characteristics of Camellia oleifera Abel. Seedlings. Forests. 2018; 9: 784. 10.3390/f9120784.

